# Self-Regulation of Candida albicans Population Size during GI Colonization

**DOI:** 10.1371/journal.ppat.0030184

**Published:** 2007-12-07

**Authors:** Sarah Jane White, Ari Rosenbach, Paul Lephart, Diem Nguyen, Alana Benjamin, Saul Tzipori, Malcolm Whiteway, Joan Mecsas, Carol A Kumamoto

**Affiliations:** 1 Department of Molecular Biology and Microbiology, Tufts University, Boston, Massachusetts, United States of America; 2 Division of Infectious Diseases, Tufts University, Grafton, Massachusetts, United States of America; 3 Genetics Group, Biotechnology Research Institute, National Research Council, Montreal, Quebec, Canada; Johns Hopkins University School of Medicine, United States of America

## Abstract

Interactions between colonizing commensal microorganisms and their hosts play important roles in health and disease. The opportunistic fungal pathogen Candida albicans is a common component of human intestinal flora. To gain insight into C. albicans colonization, genes expressed by fungi grown within a host were studied. The *EFH1* gene, encoding a putative transcription factor, was highly expressed during growth of C. albicans in the intestinal tract. Counterintuitively, an *efh1* null mutant exhibited increased colonization of the murine intestinal tract, a model of commensal colonization, whereas an *EFH1* overexpressing strain exhibited reduced colonization of the intestinal tract and of the oral cavity of athymic mice, the latter situation modeling human mucosal candidiasis. When inoculated into the bloodstream of mice, both *efh1* null and *EFH1* overexpressing strains caused lethal infections. In contrast, other mutants are attenuated in virulence following intravenous inoculation but exhibited normal levels of intestinal colonization. Finally, although expression of several genes is dependent on transcription factor Efg1p during laboratory growth, Efg1p-independent expression of these genes was observed during growth within the murine intestinal tract. These results show that expression of *EFH1* regulated the level of colonizing fungi, favoring commensalism as opposed to candidiasis. Also, different genes are required in different host niches and the pathway(s) that regulates gene expression during host colonization can differ from well-characterized pathways used during laboratory growth.

## Author Summary

Although the fungus Candida albicans commonly colonizes the human gastrointestinal tract as a commensal, the organism is also an opportunistic pathogen, responsible for a wide range of infections in immunocompromised persons. While numerous studies of infection have been conducted, few studies have analyzed the commensal state. The studies described here analyze C. albicans cells colonizing the intestinal tract of immunocompetent mice in the absence of disease, a model for commensalism. Results showed that expression of the putative transcription factor Efh1p by cells colonizing the intestinal tract was relatively high, but paradoxically, expression of Efh1p was associated with lower colonization. Efh1p had no detectable effect on the ability of C. albicans to cause lethal disseminated infection in mice. In contrast, Rbt1p and Rbt4p, two proteins of poorly defined function required for normal disseminated infection, were not required for intestinal colonization. These results argue that the commensal state is distinct from the pathogenic state and that different factors are important in different states. Also, the regulation of expression of genes *RBT1*, *RBT4*, and *ECE1* during intestinal colonization differed from their well-characterized regulation during laboratory growth. Further studies of commensal colonization are needed to understand this important stage of C. albicans biology.

## Introduction

The opportunistic fungal pathogen Candida albicans colonizes its host long before disease arises. In humans, C. albicans colonization of the oral cavity is detected in most infants by the age of one month [1]. The majority of adults are detectably colonized in the intestinal tract by C. albicans [2]. Colonization is believed to persist for long periods of time [2], and in this situation, C. albicans is primarily nonpathogenic. However, if the host becomes immunocompromised, disease caused by dissemination of commensal organisms from the intestinal tract can occur. For example, disseminated candidiasis occurs in neutropenic patients, and Candida spp are among the most common organisms isolated from the blood of hospitalized patients [3]. In AIDS patients, oropharyngeal candidiasis (OPC) is a common opportunistic infection [4]. Despite the importance of commensal organisms as the source of infection, little is known about C. albicans factors that influence intestinal colonization.

A growing body of literature indicates that the interplay between commensal organisms and the host GI tract is characterized by reciprocal regulatory interactions. In addition to a role in nutrition, normal flora are required for proper development of the intestinal capillary network as well as Peyer's patches and other components of the intestinal immune system [5]. Normal flora stimulate host toll-like receptors, and these interactions are important for regulation of the inflammatory response. In the absence of toll-like receptors, dysregulation of the inflammatory response with concomitant damage to the GI tract occurs [6]. Thus, the flora make important contributions to the health of the host.

The host also influences its flora. Bulk movement of material through the GI tract regulates populations of commensal organisms [7], and host secretions and immune effectors play key roles in regulating colonization and determining the composition of the flora [8]. Therefore, reciprocal interactions between the commensal flora and the host maintain the balance between overexuberant inflammation and uncontrolled growth of microorganisms.

To gain insight into the activities of C. albicans that are important for host colonization or disease, studies of C. albicans gene expression during interaction with a host or host cells have been pursued. Microarray studies analyzing interactions between C. albicans and cultured immune cells have detected dramatic changes in C. albicans metabolism and stress responses [9–11]. For example, genes encoding enzymes of the glyoxylate cycle are highly expressed in phagocytosed fungal cells, and isocitrate lyase is important for systemic virulence [9]. A study of antigens expressed during oral infection revealed that components of a MAP kinase signal transduction pathway were expressed [12]. In addition, the Not5 protein is expressed during oral infection and is required for systemic virulence. Recent analysis of C. albicans cells invading host parenchymal tissue revealed changes in expression of numerous genes, demonstrating that invading cells experience metabolic changes and initiate responses to stresses such as iron limitation [13].

The goals of this study were to identify C. albicans genes that were expressed during growth within a host and to compare the genetic requirements for infection and intestinal colonization. Therefore, genes expressed in C. albicans cells associated with oral infection or in cells growing in the intestinal tract were identified, and the ability of mutants lacking some of the genes to colonize the murine intestinal tract or to produce disease was characterized. Mutants lacking the *EFH1* gene, encoding a putative transcription factor [14], colonized the intestinal tract at higher levels than wild-type (WT) organisms. Strains engineered to overexpress *EFH1* exhibited reduced levels of intestinal colonization and reduced colonization of the oral cavity of immunodeficient (athymic) mice. Therefore, *EFH1* is important for determining the population size of C. albicans during colonization. Despite its importance during intestinal colonization, *EFH1* did not affect virulence in the disseminated infection model or cellular physiology under laboratory conditions. In contrast, *RBT1*, encoding a putative cell wall protein, and *RBT4*, encoding a possible secreted protein, are required for normal virulence in the disseminated infection model [15] but are not required for normal intestinal colonization, despite their high expression during growth in the intestinal tract. Therefore, genes that influence commensal colonization can be distinct from genes that are required for virulence.

## Results

### C. albicans Gene Expression during Growth within an Immunosuppressed Mammalian Host

To initiate studies on C. albicans genes required for infection or colonization, preliminary microarray analysis of gene expression in C. albicans cells recovered from sites of oral infection was performed. WT C. albicans cells were orally inoculated into germ-free newborn piglets as described [16]. Following immunosuppression with cyclosporine A and methylprednisolone for 7–10 d, the animals developed dramatic thrush and esosphagitis, and C. albicans plaques were visible in the esophagus at necropsy. C. albicans cells were scraped from the tongues of two immunosuppressed, gnotobiotic (IGB) piglets and from the esophagus of one of them and were used for microarray analysis as described in Materials and Methods. Because the majority of spots exhibited poor hybridization with samples from the oral infections, a comprehensive analysis of differential gene expression could not be performed. However, there were several candidate genes more highly expressed in samples taken from infected piglets than in reference laboratory-grown cells.

To confirm that candidate genes from the microarray studies exhibited increased expression in mammalian hosts compared to log phase laboratory-grown cells, quantitative real-time reverse transcriptase PCR (qRT-PCR) was performed. For the analysis, RNA was prepared from C. albicans cells scraped from the tongue, esophagus, or roof of the mouth of two IGB piglets or recovered from the contents of the large intestine of one IGB piglet. cDNA prepared from the RNA was used as the template for qRT-PCR amplification as described in Materials and Methods. Results were normalized using *C. albicans ACT1* (encoding actin) and are shown relative to the reference sample of log phase, laboratory-grown cells that were used for the microarrays.

Seven genes that exhibited relatively high expression in C. albicans cells recovered from the oral cavity or intestinal tract were identified, including *EFH1*, *YHB5*, and *ECE1* (Table 1), which became the subjects of this study. *EFH1* encodes a paralog of the well-studied transcription factor Efg1p [17]. Efg1p regulates several morphological transitions as well as the expression of hyphal and virulence genes [14,18–21]. However, the absence of Efh1p does not lead to a pronounced phenotype during laboratory growth [14], and thus, its function is not understood. *YHB5* (orf19.3710) encodes a flavohemoglobin; its paralog, *YHB1*, is induced by growth in the presence of nitric oxide and is important for resistance to nitrosative stress [22,23]. *YHB5*, by contrast, is not induced during laboratory growth in nitric oxide [22]. *ECE1*, a gene that influences adhesion [24], but whose function is poorly understood, is highly expressed by cells that are growing in the hyphal form (filamentous cells lacking constrictions at their septa) [21,24,25]. *ECE1* is also highly expressed during invasion of host tissue [13].

**Table 1:**
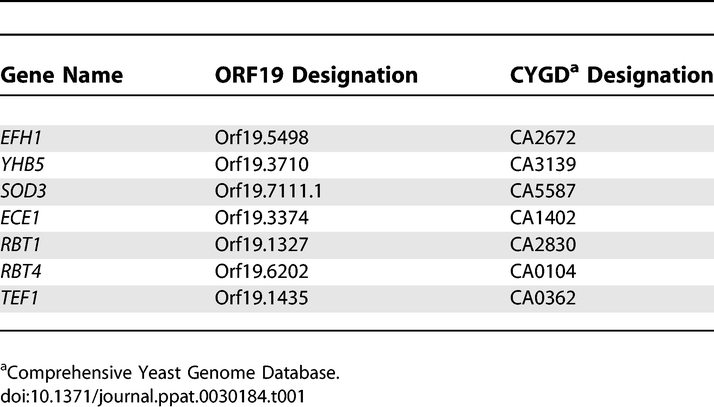
Gene Designations

In addition*,* other genes expressed under laboratory conditions that resulted in increased *ECE1* or *EFH1* expression (hyphal growth and post-exponential phase, respectively) were analyzed. *RBT1* and *RBT4*, genes of poorly understood function that are expressed in hyphal cells [15], and *SOD3* (orf19.7111.1), encoding a manganese-containing superoxide dismutase that is expressed in post-exponential phase [26], were studied. Finally, a control housekeeping gene, *TEF1* (encoding translation elongation factor 1-alpha), was analyzed in some experiments.

**Figure 1:**
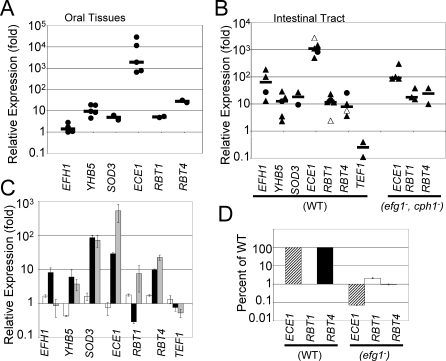
Expression of C. albicans Genes Expression of genes of interest in cDNA preparations was determined by qRT-PCR as described in Materials and Methods. Results were normalized using actin expression and are expressed relative to a reference sample of laboratory-grown log phase cells. (A) Gene expression in C. albicans cells recovered from the oral cavities or esophagi of IGB piglets. Closed circles, WT C. albicans cells (SC5314) recovered from the tongue, roof of the mouth, or esophagus; bars, geometric means. (B) Gene expression in C. albicans cells recovered from the intestinal tract. Closed circles, WT C. albicans cells (SC5314) recovered from the large intestine of an IGB piglet; closed triangles, WT (SC5314 or DAY185), or mutant (*efg1 cph1* double null) C. albicans cells recovered from the cecum of Swiss Webster mice; open triangles, WT C. albicans (SC5314) recovered from the ileum of Swiss Webster mice; bars, geometric means. (C) Gene expression during laboratory growth. White bars, log phase C. albicans grown in rich medium at 37 °C; black bars, post-exponential phase C. albicans grown in rich medium at 37 °C; grey bars, hyphal C. albicans grown in RPMI-serum at 37 °C; error bars, standard deviation. (D) Comparison of gene expression in WT C. albicans (DAY185) and in an *efg1* deletion mutant grown in RPMI-serum at 37 °C. Cross-hatched bars, relative *ECE1* expression; white bars, relative *RBT1* expression; black bars, relative *RBT4* expression; error bars, standard deviation.

The qRT-PCR results for the genes of interest are shown in Figure 1. In C. albicans cells recovered from oral infection, five of the genes were highly expressed relative to their expression in log phase laboratory-grown cells (Figure 1A). The sixth gene, *EFH1*, was not convincingly upregulated in cells recovered from oral lesions. In cells recovered from the piglet intestine, *EFH1* and the five other genes showed relatively high expression (Figure 1B, closed circles). Since only one piglet intestinal tract sample was studied, these initial data on gene expression in the intestinal tract were not definitive. Further studies in mice corroborated the results, as described below.

To compare expression during growth in the host with expression during growth in laboratory conditions that might mimic certain host parameters, cells were grown to a post-exponential phase (rich medium 37 °C, 3 d) or under conditions that resulted in formation of hyphae (serum-containing medium, 37 °C), and compared to log phase cells (rich medium 37 °C). Expression was measured by qRT-PCR as above. Figure 1C shows that most of the genes of interest were more highly expressed in post-exponential phase than in log phase and all genes except for *EFH1* were relatively highly expressed in hyphae. In contrast, expression of the housekeeping gene *TEF1* was not markedly induced under these conditions. Thus, several genes were relatively highly expressed during growth within multiple tissues in the host and in either post-exponential phase or hyphal growth in the laboratory.

### Gene Expression during Intestinal Colonization Is Not Dependent on Host Immunosuppression

For further studies of the interaction between C. albicans and a mammalian host, we analyzed gene expression during colonization of immunocompetent Swiss Webster mice. Following oral inoculation of antibiotic-treated Swiss Webster mice, commensal colonization persisted for several weeks as previously described, e.g., [27–29]. No symptoms of disease were observed in the colonized mice.

To determine whether the genes of interest were expressed during commensal intestinal colonization, gene expression was measured by qRT-PCR using RNA prepared from C. albicans cells recovered from the cecum or ileum of colonized mice. Expression normalized to *ACT1* is expressed relative to the same reference sample as above. As shown in Figure 1B (closed triangles), all six genes of interest were expressed at relatively high levels in C. albicans cells recovered from the mouse cecum in contrast to the housekeeping gene *TEF1*. Expression was very similar to the expression observed in C. albicans cells recovered from the IGB piglet intestinal tract. Therefore, the expression of these genes was not dependent on immunosuppression of the host. In addition, to compare expression in a different part of the intestinal tract, expression of three genes was characterized in cells recovered from the ileum. *ECE1* and *RBT4* were relatively highly expressed and *RBT1* was slightly increased. In summary, five of the six genes were relatively highly expressed during growth within multiple tissues of immunosuppressed and immunocompetent hosts. *EFH1* exhibited a distinct pattern of expression as it was relatively highly expressed in C. albicans cells recovered from the intestinal tract but not from sites of oral infection (esophagus or tongue lesions).

### Uncharacterized Regulatory Pathways Control Gene Expression during Growth in the Intestinal Tract

**Figure 2:**
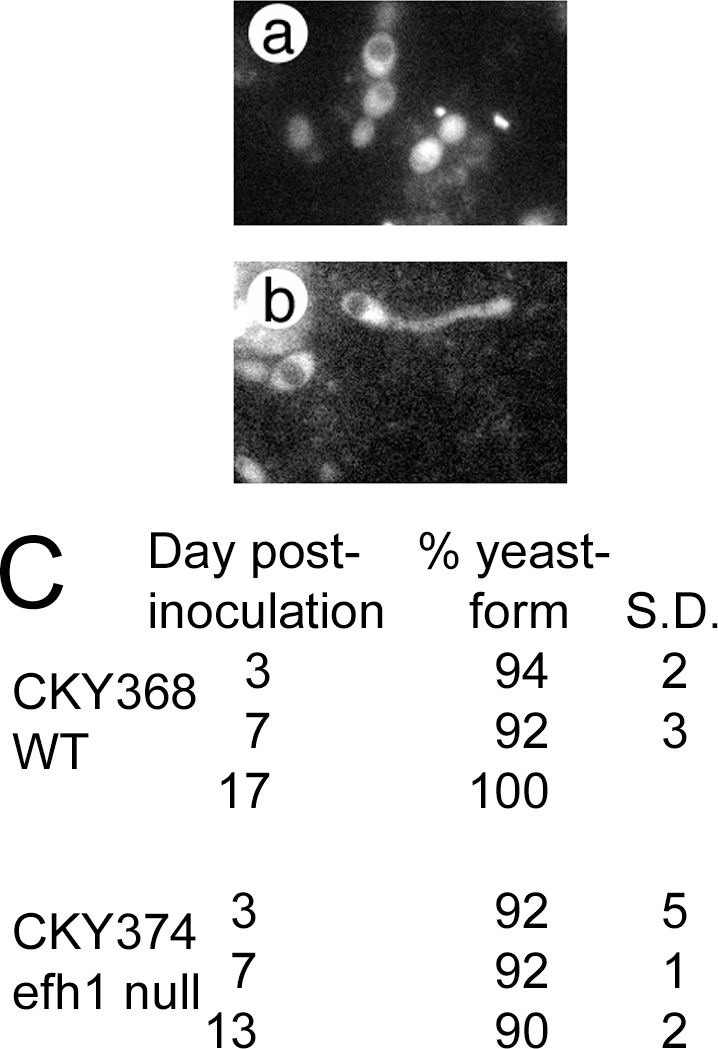
C. albicans Cells Colonizing the Murine Intestinal Tract Are Predominantly in the Yeast Form (A and B) Contents of the ileum of mice inoculated with C. albicans strain CKY368, WT, GFP-expressing, day 3 post-inoculation (A) or strain CKY374, *efh1* null mutant, GFP-expressing day 13, post-inoculation (B). Fluorescence micrographs show the morphology of yeast-form cells (A) or hyphal-form cells (B). (C) Quantitation of cellular morphology.

Because most of the genes of interest were highly expressed in laboratory-grown hyphae (Figure 1C), it was of interest to determine the morphology of the colonizing cells. C. albicans cells expressing yeast-enhanced green fluorescent protein (yEGFP) [30] were orally inoculated into mice by gavage. Three, seven, or 17 days post-inoculation, the contents of the ileum were recovered, and the C. albicans cells were observed by fluorescence microscopy. In all samples, the vast majority of the fluorescent cells (>90% +/− 3% standard deviation) exhibited yeast cell morphology (Figure 2). Because the cecum is anaerobic, it was not possible to visualize GFP in this organ, so phase contrast microscopy was used to visualize organisms. As with the ileum, the majority of fungal cells detected in cecum contents exhibited the yeast-cell morphology (71% +/− 10% standard error of the mean).

Interestingly, cells recovered from the ileum expressed higher levels of *ECE1* and *RBT4* than laboratory-grown yeast cells and these levels were comparable to laboratory-grown hyphal cells. This observation suggested that the yeast-form cells recovered from the ileum, rather than the rare hyphal-form cells, were expressing high levels of *ECE1* and *RBT4*. Therefore, gene expression during growth within the host differed from the characterized expression of *ECE1* and *RBT4* under laboratory conditions.

To compare gene expression during laboratory growth and host growth further, the role of the morphogenesis-regulating transcription factor Efg1p was studied. In the laboratory, expression of *ECE1*, *RBT1*, and *RBT4* in hyphae is dependent on Efg1p (Figure 1D and [31,32]). Expression is similarly reduced in a double mutant lacking a second partially redundant transcription factor, Cph1p [33] and Efg1p (unpublished data and [32]). Therefore, the effect of deletion of both *EFG1* and *CPH1* on expression of these three genes during growth in the intestinal tract was studied. Strain CKY138 (*efg1 cph1* double null mutant) was orally inoculated into mice by gavage. RNA was prepared from cells recovered from the intestinal tract and expression of *ECE1*, *RBT1*, and *RBT4* was measured by qRT-PCR. Unexpectedly, double null mutant C. albicans cells recovered from the intestinal tract expressed *ECE1*, *RBT1*, and *RBT4* (Figure 1B). Thus, there was substantial Efg1-, Cph1p-independent expression of *ECE1*, *RBT1*, and *RBT4* during growth within the intestinal tract. These observations demonstrate that an uncharacterized regulatory pathway(s) controls the expression of these genes during growth in the intestinal tract, underscoring the importance of studying C. albicans physiology during growth within a host.

### Expression of *EFH1* Results in Reduced Colonization of the Intestinal Tract

Five of the six genes of interest were expressed at elevated levels in all samples recovered from the host, while *EFH1* expression was elevated in cells recovered from the intestinal tract but not from the tongue or esophagus. To determine whether *EFH1* performs a function during colonization of the intestinal tract, *efh1* null mutants and *EFH1* reconstituted strains were constructed.

As previously reported [14], deletion of *EFH1* did not result in defects in growth under laboratory conditions. Hyphal formation by the mutant strain was also normal and the *efh1* null mutant was indistinguishable from WT in post-exponential phase stress resistance (heat shock at 55 °C or menadione treatment; unpublished data). The mutant strain grew well anaerobically in defined medium [34] (I. Soltero and C. A. Kumamoto, unpublished data). Therefore, as noted previously [14], the *efh1* deletion appeared to have minimal effects on the physiology of laboratory-grown cells.

**Figure 3:**
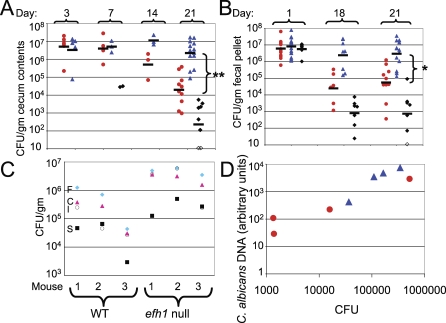
Deletion of *EFH1* Alters Murine Intestinal Colonization WT, *efh1* deletion mutant, or *EFH1* reconstituted null mutant were orally inoculated by gavage into Swiss Webster mice. At various days post-inoculation, the amounts of C. albicans in fecal pellets and in organs of the intestinal tract were measured. (A) CFUs per gram of cecum contents from mice sacrificed on the indicated days post-inoculation. Each symbol represents a sample from a different mouse. (Composite results from several experiments.) Red circles, WT C. albicans strain DAY185 (23 mice); blue triangles, *efh1* deletion mutant strain CKY366 (24 mice); black diamonds, *EFH1* reconstituted strain, CKY373 (ten mice); open symbols, no colonies detected; bars, geometric means. *p*-Value was determined using the *t* test with log transformed data. ** indicates *p* < 0.000005. (B) CFUs per gram of fecal pellet. Mice were sampled repeatedly and each symbol represents a sample from a different mouse. Different numbers of mice are shown on different days because some mice were sacrificed earlier and some mice were sampled on different days. Red circles, WT C. albicans strain DAY185 (ten mice); blue triangles, *efh1* deletion mutant strain CKY366 (12 mice); black diamonds, *EFH1* reconstituted strain, CKY373 (seven mice); open symbols, no colonies detected; bars, geometric means. *p*-Value was determined using the *t* test with log transformed data. * indicates *p* < 0.0003. (C) Correlation between CFUs measured in several organs and in fecal pellets. Light blue diamonds, fecal pellets (labeled F); pink triangles, cecum contents (labeled C); open circles, ileum contents (labeled I); squares, stomach contents (labeled S). CFU/gm of cecum contents, stomach contents, and fecal pellets correlated well, while the CFU/gm ileum was more variable. (D) Mice were colonized with WT strain DAY185 (red circles) or *efh1* null mutant CKY366 (blue triangles) or were not inoculated with C. albicans (not shown). Mice were sacrificed on day 21 post-inoculation, CFUs from cecum contents were determined by plating, and C. albicans DNA in cecum contents was quantified by qPCR as described in Materials and Methods. The correlation between CFUs in 70 mg of pelleted cecum contents (*x*-axis) and DNA (in arbitrary units) determined by qPCR (*y*-axis) is shown. Each symbol represents a different mouse.

When WT C. albicans, *efh1* null mutant, or *EFH1* reconstituted null mutant cells were orally inoculated by gavage into immunocompetent mice, intestinal tract colonization was established. As shown in Figure 3, WT C. albicans colonized the intestinal tract and was detectable in the contents of the cecum up to 3 wk post-inoculation (Figure 3A, closed circles). Colonization was also detectable in the ileum, stomach, and fecal pellets (Figure 3B and 3C); analysis of these samples from three individual mice revealed a correlation in colonization levels in different organs (Figure 3C).

To determine the effect of coprophagy on colonization, an uninoculated mouse was introduced into a cage with three to four inoculated mice, 24 h post-inoculation. Fecal pellets from the uninoculated mouse were initially positive for C. albicans, but the levels of C. albicans declined very rapidly, and 8 d post-introduction into the cage were at least 50-fold below the geometric mean for the inoculated mice. Therefore, consistent with previous studies [29], the contribution to C. albicans titers due to coprophagy was minimal at later time points.

Surprisingly, two independently isolated *efh1* null mutants colonized the murine intestinal tract at higher levels (Figure 3A, blue triangles) than WT C. albicans (Figure 3A, red circles). In the cecum at day 21 post-inoculation, the geometric mean for the *efh1* null mutant was 100-fold higher than that for WT C. albicans (*p* < 0.000005 by *t* test). For both the *efh1* null mutant and WT strains, C. albicans associated with the cecum wall represented a small fraction of the total cecum-associated C. albicans. Analysis of C. albicans in fecal pellets demonstrated that, initially, colonization by the mutant was similar to colonization by WT C. albicans (Figure 3B) but at later time points, e.g., day 21 post-inoculation, colonization by the *efh1* null mutant persisted at higher levels than the WT strain (Figure 3B, *p* < 0.0003 by *t* test). Enhanced colonization was also observed in the ileum and stomach (Figure 3C and unpublished data).

As a control to show that the differences in colony forming units (CFUs) recovered from WT or *efh1* mutant–containing ceca truly reflected differences in the numbers of fungal cells rather than preferential recovery of mutant cells, an alternative method of quantification was used. DNA was extracted from cecum contents and the amount of C. albicans genomic DNA was determined by quantitative PCR (qPCR). The results showed that the amounts of C. albicans DNA recovered from cecum contents containing WT or *efh1* null mutant cells (in arbitrary units) correlated with the CFUs determined by plating (Figure 3D), and the average ratio of the amount of DNA/CFU was the same for the WT and *efh1* null mutant strains. Therefore, differences in CFUs reflected differences in the numbers of colonizing C. albicans.

To demonstrate that the enhanced colonization reflected the absence of Efh1p, *EFH1* was added back to the deletion mutant under control of a strong promoter to ensure expression of the reintroduced gene. Introduction of the ectopically controlled *EFH1* to the mutant strain resulted in reduced colonization (Figure 3A, black diamonds), indicating that the level of *EFH1* in the strain determined colonization levels. For unknown reasons, introduction of *EFH1* at its native locus did not result in full complementation (unpublished data), as has been observed by others for other genes, e.g., [15]. These studies demonstrate that in the absence of Efh1p, colonization of the intestinal tract was enhanced. Thus, paradoxically, expression of *C. albicans EFH1* during commensal colonization of the intestinal tract resulted in reduced colonization.

To observe the morphology of *efh1* null mutant cells, GFP-expressing *efh1* null mutants were orally inoculated into mice by gavage. On day 3 or day 13 post-inoculation, ileum contents were collected and organisms were visualized by observing green fluorescence. As observed for WT *C. albicans*, the vast majority (>91% +/− 1% standard deviation) of cells exhibited yeast-form morphology (Figure 2).

To detect dissemination from the intestinal tract in mice, the kidneys, liver, and spleen were homogenized and cultured. With both *efh1* null mutant and WT, all samples were either negative or contained very few organisms (Table 2). Therefore, there was no evidence of high-level colonization of deep organs, indicating that C. albicans was not escaping from the intestinal tract.

**Table 2:**
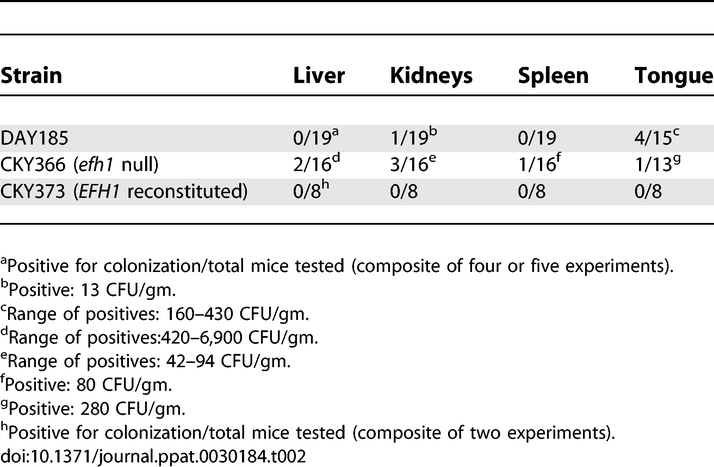
Organ Colonization

Colonization of the tongue of mice was analyzed to determine whether a significant level of oral candidiasis was occurring. Colonization was generally not detectable, although an occasional mouse exhibited below 10^3^ CFU/gm tongue tissue (Table 2). No consistent differences in colonization by WT and mutant organisms were observed. Therefore, there was no evidence of either mucosal or systemic disease in these immunocompetent mice.

To determine the levels of residual bacteria remaining after antibiotic treatment, ileum and cecum homogenates were cultured under aerobic or anaerobic conditions on rich media. The ranges of bacteria levels were very similar for uninoculated mice and for mice inoculated with all of the C. albicans strains described above (unpublished data).

### *EFH1* Overexpression Reduces Intestinal Colonization

Since reduction of Efh1p by deletion resulted in enhanced colonization, the effect of increased expression of Efh1p was tested using an *EFH1* ^+^ strain carrying a third copy of *EFH1* expressed from the strong *ADH1* promoter. The growth rate of the *EFH1* overexpressing strain was close to WT during laboratory growth in CM medium at 37 °C (WT doubling time 78 min; overexpressing strain, 82 min), and high levels of *EFH1* transcript were produced under these conditions (unpublished data). In rich medium, the overexpressing strain produced yeast-form cells while in certain minimal media the mutant produced pseudohyphae, consistent with previous results [14].

**Figure 4:**
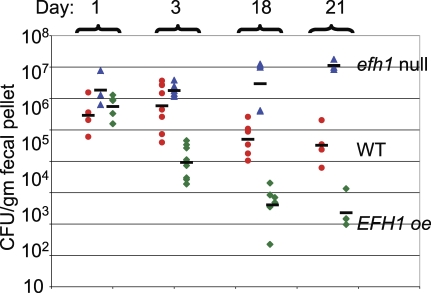
Attenuated Colonization of Mice by an *EFH1* Overexpressing Strain Cells of WT strains CKY363 or DAY185, *efh1* deletion mutant strain CKY366, and *EFH1* overexpressing strain CKY364 were orally inoculated by gavage. At various days post-inoculation, fresh fecal pellets were recovered from inoculated mice and the amount of C. albicans per gm was measured. Red circles, CKY363 or DAY185 (WT) (seven mice); blue triangles, *efh1* deletion mutant strain CKY366 (six mice); dark green diamonds, CKY364 (*EFH1* overexpressing) (eight mice); bars, geometric means. Composite results of two experiments are shown. Different numbers of mice are shown on different days because some mice were sacrificed earlier and some mice were sampled on different days.

When the overexpressing mutant was orally inoculated into mice by gavage, colonization was initially similar to that of WT C. albicans (Figure 4). Subsequently, however, colonization by the overexpressing strain declined more rapidly than colonization by the WT strain, and at days 18 and 21 post-inoculation, the geometric mean for the overexpressing strain was more than 100-fold lower than that of the WT strain (*p* < 0.002 using *t* test). This result is consistent with that of the *EFH1* reconstituted null mutant (Figure 3). Therefore, high expression of *EFH1* attenuated colonization, while deletion of *EFH1* resulted in enhanced colonization, demonstrating that Efh1p is a regulator of the level of colonization during growth of C. albicans in the murine intestinal tract.

### Effects of *EFH1* on Disease

In piglet oral lesions, expression of *EFH1* was low relative to its expression in cells growing within the intestinal tract. These findings suggest that when infection occurs, the negative effects of *EFH1* on host colonization are relieved by lowering *EFH1* expression. Therefore, ectopic *EFH1* overexpression might reduce the ability of C. albicans to cause disease. To test this model, the behavior of an *EFH1* overexpressing strain was analyzed in two different animal models.

In immunocompromised patients, C. albicans causes mucosal infections, such as OPC. Paralleling the susceptibility of AIDS patients to OPC, mice lacking T cells show an enhanced susceptibility to oral colonization by C. albicans [35]. To determine whether *EFH1* overexpression would influence colonization in the oral cavities of immunocompromised mice, competition experiments were performed, in which mixtures of *EFH1* overexpressing and genetically marked WT C. albicans cells were inoculated directly into the oral cavities of athymic mice by swabbing. The marked WT strain carried the *SAT1* nourseothricin resistance gene [36] under control of the maltase promoter. To monitor the ability of the two strains to persist in the oral cavity relative to one another, the oral cavities were swabbed at various times post-inoculation, and the ratio of the two strains was determined by replica plating. The competitive index (CI) was determined by dividing the ratio of Nou^S^ and Nou^R^ strains at a particular time point by the ratio in the inoculum.

**Figure 5:**
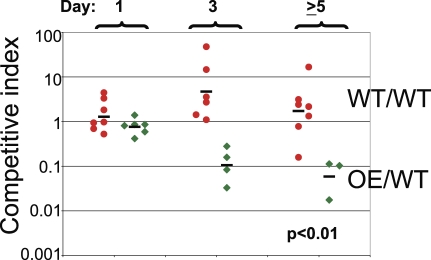
*EFH1* Overexpression Reduces Colonization of the Oral Cavity in Immunodeficient Mice Cells of strains CKY363 (WT) or CKY364 (*EFH1* overexpressing, OE) were mixed in a 1:1 ratio with strain RMIS1 (Nou^R^ marked WT) and introduced into the oral cavities of nude mice by swabbing. On various days post-inoculation, the oral cavities were sampled by swabbing. Swabs were rubbed on YPD SA plates and the colonies were replica-plated to YPSnourseothricin to determine the ratio of Nou^S^ to Nou^R^ colonies. The CI is defined as the ratio of Nou^S^/Nou^R^ colonies at time x, divided by their ratio in the inoculum. Each symbol represents a sample taken from an individual animal. Composite results of two experiments are shown. Red circles, CKY363 (WT) mixed with RMIS1 (seven mice); dark green diamonds, CKY364 (*EFH1* overexpressing) mixed with RMIS1 (six mice). Day ≥5 includes samples taken at either day 5 or day 6. Samples that yielded very few colonies (<10) were not included on the graph. A statistically significant difference (*p* < 0.01 by *t* test) was observed at day ≥5.

When unmarked WT and marked WT strains were mixed and inoculated, the geometric means for the CI remained above 1 throughout the time course, indicating a slight competitive advantage for the unmarked WT strain (Figure 5). For the competition between the Nou^S^ *EFH1* overexpressing strain and the Nou^R^ WT strain, the CI was close to 1 on day 1 post-inoculation, but declined thereafter, demonstrating that the *EFH1* overexpressing strain exhibited a competitive disadvantage relative to the WT strain (Figure 5). The difference in CI for WT and *EFH1* overexpressing strain was statistically significant (*p* < 0.01 by *t* test). At longer times, few colonies were obtained, precluding the accurate measurement of ratios. Following 24 h of laboratory growth in CM medium at 37 °C, the CI for the overexpressing strain relative to the marked WT was 1.1 (average of two determinations), showing a similar growth rate for the strains under these conditions. Therefore, these data show that forced high expression of *EFH1* reduces colonization in the oral cavity of immunodeficient mice. In immunocompetent BALB/c mice, oral colonization was rapidly lost (unpublished data), and it was not possible to measure ratios.

To determine whether *EFH1* was important in other host niches, the ability of the *efh1* null mutant and *EFH1* overexpressing strain to cause lethal infection in a disseminated candidiasis model was tested. When inoculated intravenously in mice, both the *efh1* null mutant and the *EFH1* overexpressing strain retained the ability to cause lethal infections. The mean survival time for mice inoculated with WT C. albicans was 5 ± 3 d (23 mice). For the *efh1* null mutant, the mean survival time was 5 ± 3 d (16 mice) and for the *EFH1* overexpressing strain, the mean survival time was 7 ± 3 d (seven mice) (composite results from at least two experiments). Therefore, both the *efh1* null mutant and the *EFH1* overexpressing strain were virulent in this model.

### *rbt1* and *rbt4* Mutants Colonize the Murine Intestinal Tract at WT Levels while the *ece1* Mutant Is Attenuated

**Figure 6:**
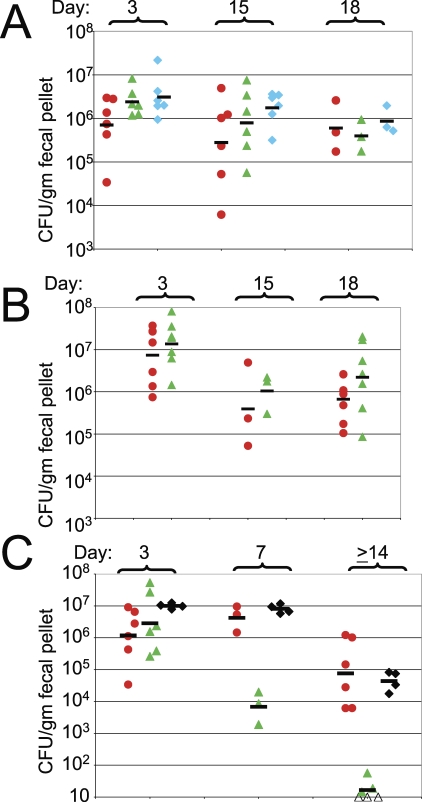
*rbt1* and *rbt4* Mutants Colonize the Murine Intestinal Tract at WT Levels Cells of WT strains DAY185 or F2U, *rbt1* deletion mutant strain BCa 7–4, *rbt4* null mutant strain BCa 11–3, *ece1* null mutant CAW19–1, CKY362 (*ECE1* reconstituted), and CKY376 (*yhb1 yhb5* double null mutant) were orally inoculated by gavage. At various days post-inoculation, fresh fecal pellets were recovered from inoculated mice and the amount of C. albicans per gm was measured. Composite results of two experiments are shown. Different numbers of mice are shown on different days because some mice were sacrificed earlier and some mice were sampled on different days. (A) Red circles, DAY185 or F2U (WT) (six mice); green triangles, *rbt1* deletion mutant strain BCa 7–4 (six mice); blue diamonds, *rbt4* deletion mutant strain Bca 11–3 (six mice); bars, geometric means. (B) Red circles, DAY185 (WT) (six mice); green triangles, *yhb1 yhb5* double null mutant strain CKY376 (six mice); bars, geometric means. (C) Red circles, DAY185 or F2U (WT) (six mice); green triangles, *ece1* deletion mutant strain CAW19–1 (six mice); black diamonds, *ECE1* reconstituted mutant strain CKY362 (four mice); bars, geometric means. Open symbols, no colonies detected. Day ≥ 14 indicates samples taken on day 14 or day 15.

The ability of mutants lacking some of the other genes of interest to colonize the intestinal tract of mice was analyzed. The *rbt1* null mutant (Figure 6A), *rbt4* null mutant (Figure 6A), and *yhb1 yhb5* double null mutant (Figure 6B) colonized the intestinal tract at close to WT levels. In contrast, the *ece1* mutant exhibited an attenuated colonization phenotype, which was reversed when the WT *ECE1* gene was added back to the mutant strain (Figure 6C). Therefore, two of the six genes tested in this study that were relatively highly expressed during growth in the intestinal tract, *EFH1* and *ECE1*, influenced the ability of C. albicans to colonize.

## Discussion

In this communication, several genes that were relatively highly expressed during growth within a host (host-growth genes) were characterized. The host-growth genes differ in their importance for intestinal colonization and systemic virulence demonstrating that different factors control host-pathogen interactions in different sites. The genes *RBT1* and *RBT4* are required for C. albicans to cause lethal infection following intravenous inoculation and for invasive infection of the cornea [15]. However, these genes are not required for colonization of the intestinal tract. In contrast, the *C. albicans EFH1* gene regulates intestinal colonization but not systemic infection. Thus, some genes are required in one niche but not in another. Intestinal colonization is an essential stage in the life cycle of C. albicans because the organism is not thought to have an environmental reservoir. Therefore, optimal intestinal colonization is crucial for survival of the organism, and it is not surprising that the organism possesses genes that regulate colonization in this niche.

*EFH1* was relatively highly expressed by C. albicans cells growing in the mammalian intestinal tract, but paradoxically, expression of *EFH1* was associated with lower colonization. Ectopic overexpression of *EFH1* resulted in very poor colonization; this latter finding probably reflects a true difference in colonization, because for in vitro–grown *EFH1* overexpressing cells, the ratio of DNA content (determined by qPCR) to CFUs (measured by plating) was less than 2-fold less than the ratio obtained for WT *C. albicans* (unpublished data). In this niche, therefore, expression of *EFH1* had a negative effect on population size. Expression of Efh1p may affect the interactions between colonizing C. albicans and the cells of the intestinal tract, resulting in changes such as altered adherence or altered signaling to the immune system. By reducing colonization, expression of *EFH1* may also reduce the likelihood of C. albicans infection, favoring commensal colonization as opposed to candidiasis.

The effects of Efh1p on colonization are reversible, because expression of *EFH1* was regulated during growth within the host (lower expression in cells recovered from OPC lesions than in cells recovered from the intestinal tract). Therefore, the negative effects of Efh1p that reduce colonization are inactivated during oral infection. Since forced expression of *EFH1* reduced colonization of the oral cavity in an immunodeficient host, repression of *EFH1* expression during active infection may be an important step in the progression from benign colonizer to active invader on mucosal surfaces.

Since *EFH1* encodes a putative transcription factor, its effects on colonization are probably indirect. Most likely, deletion of *EFH1* alters the expression of other genes, some of which directly influence colonization. Under laboratory conditions, *EFH1* did not strongly affect the expression of the collection of genes studied in this report (unpublished data). However, in previous studies, a laboratory-grown *EFH1* overexpressing strain was shown to have altered expression of several cell surface proteins [14], which may play roles in colonization.

The genes studied here were relatively poorly expressed in laboratory-grown log phase cells but showed expression in laboratory-grown post-exponential phase cells, suggesting that during growth within a host, C. albicans cells express some features of post-exponential phase cells. Numerous bacterial genes that play important roles governing host-pathogen interactions are expressed in post-exponential phase. For example, expression of virulence factors by organisms such as Bacillus anthracis [37], Staphylococcus aureus [38], Helicobacter pylori [39], Legionella pneumophila [40], or *Salmonella* [41] occurs in post-exponential phase. Therefore, expression of post-exponential phase genes during host interaction is a common theme for many bacterial pathogens and for the fungus C. albicans.

In the laboratory, expression of *ECE1*, *RBT1*, and *RBT4* is linked to hyphal morphogenesis. However, in cells growing within the intestinal tract, expression of these genes does not depend on Efg1p and most likely occurs in yeast cells. *ECE1*, *RBT1*, and *RBT4* were also found to be expressed in *efh1* null mutants recovered from the cecum of mice (unpublished data). Therefore, the pathway(s) responsible for the expression of these genes during growth within the host is uncharacterized. Since the genes show relatively high expression during post-exponential phase in the laboratory, there may be a post-exponential phase regulator(s) that is responsible for their expression in the host. Several regulators required for normal viability in post-exponential phase have been recently described [42]. Deeper understanding of the biology of post-exponential phase cells may reveal C. albicans activities that are important for colonization and disease.

During colonization, the population size of a microorganism reflects a balance between external forces that limit the population, such as the effects of the host immune system, and intrinsic factors, such as the ability of the organism to increase in number. The ability of commensal C. albicans to regulate its own population size through reversible expression of a negative regulator of colonization adds another layer of regulation to the interactions that take place between host and colonizer. The combined effects of these regulatory interactions maintain the balance between healthy colonization and disease.

## Materials and Methods

### Strains.

C. albicans strains are listed in Table 3. All C. albicans strains were derived from the WT clinical strain SC5314 [43], using the following genetically marked derivatives: CAI-4 [43], BWP17 [44], RM1000#2 [45], or SN100 [45].

**Table 3:**
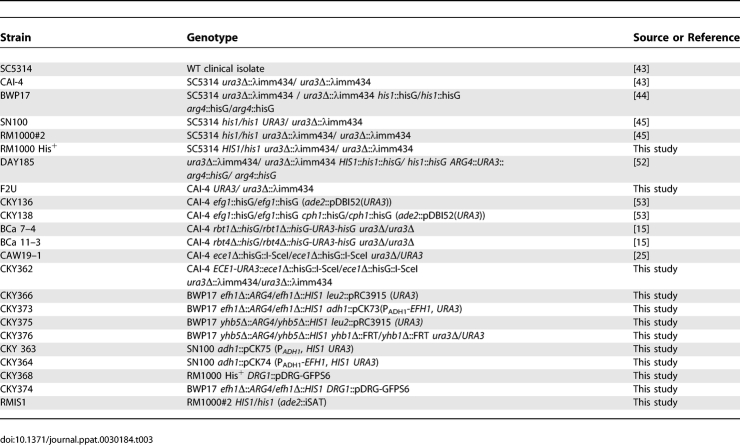
Candida albicans Strains

Strain CKY366, the *efh1* null mutant strain, was constructed by transformation of BWP17 with constructs encoding *HIS1* or *ARG4* flanked by 600 bp of sequence upstream of the *EFH1* ORF and 542 bp of sequence downstream of the *EFH1* ORF. The fragment was excised from the plasmid backbone by digestion with BslI and BsmFI for *efh1*Δ::*ARG4* or with NotI sites that were introduced at the ends of the *EFH1* sequences for *efh1*Δ::*HIS1*.

The *efh1* null mutant was transformed with pRC3915 [46] to restore *URA3* prototrophy or with pCK73 to introduce *EFH1* under control of the *ADH1* promoter*.*

To construct an *EFH1* overexpressing strain, the *EFH1* ^+^ strain SN100 (*his1/his1*) was transformed with pCK74 encoding *EFH1* under control of the *ADH1* promoter and carrying selectable marker *HIS1.*

yEGFP- [30] expressing *EFH1* ^+^ and *efh1* null mutant strains were constructed by transforming RM1000 His^+^ or the *efh1* null mutant with pDRG-GFPS6, encoding yEGFP under control of the *DRG1* promoter [47].

Strain CKY375, the *yhb5* null mutant strain, was constructed by transformation of BWP17 with constructs encoding *HIS1* or *ARG4* flanked by 496 bp of sequence upstream of the *YHB5* ORF and 355 bp of sequence downstream of the *YHB5* ORF. The desired fragments were excised from their plasmid backbones by digestion with TseI and BslI for *yhb5*Δ::*ARG4* or with NotI for *yhb5*Δ::*HIS1*. Strain CKY376, the *yhb1 yhb5* double null mutant strain, was constructed by transformation of the *yhb5* deletion mutant with a construct encoding the SAT flipper [36] with 750 bp of sequence upstream of the *YHB1* ORF and 490 bp of sequence downstream of the *YHB1* ORF. Nourseothricin-resistant (Nou^R^) transformants were screened initially by PCR. Positive candidates were plated on sucrose-containing medium to induce expression of the FLP recombinase and replica-plated to nourseothricin medium. Nou^S^ colonies were purified and subjected to a second round of transformation as above and null mutants were identified by Southern blot analysis. To generate *URA3* prototrophs, the strains were transformed with a *URA3* ^+^ fragment from plasmid pET16 [48].

The *ece1* null mutant CAW19–1 was kindly provided by B. Fonzi (Georgetown University) and the *rbt1* and *rbt4* null mutants (BCa 7–4 and Bca 11–3, respectively) were kindly provided by S. Johnson (UCSF).

To construct a genetically marked WT strain, plasmid iSAT was digested with BsgI and integratively transformed into strain RM1000#2, His^+^. The resultant strain (RM1000 iSAT) exhibits resistance to nourseothricin when grown on plates containing sucrose but poorer resistance on plates containing glucose. The iSAT construct was used because expression from the maltase promoter was expected to be low during growth within a host, minimizing possible deleterious effects due to expression of the heterologous *SAT1* gene.

### Media and growth conditions.

Standard rich media were YPD (1% yeast extract, 2% peptone, 2% glucose) or YPS (1% yeast extract, 2% peptone, 2% sucrose). Minimal dropout media (lacking uracil, histidine, arginine, or combinations) were as described previously [49]. RPMI 1640 (Sigma) with 10% bovine serum was used to promote hyphal morphogenesis. Nourseothricin (200 μg/ml) was used as described [36]. For plating contents of the intestinal tract, YPD agar medium supplemented with 50 μg/ml ampicillin and 100 μg/ml streptomycin (YPD SA) was used. For competition experiments, colonies on YPD SA were replica-plated to YPsucrose supplemented with nourseothricin.

For culturing bacteria from the intestinal tract, BHIS medium was used (3.7% Difco brain heart infusion broth, 0.5% yeast extract, 0.0015% hemin, 0.2% agar), and plates were incubated aerobically or anaerobically at 37 °C.

For gene expression studies, reference cells were grown in YPS liquid medium at 34 °C in log phase. For the experiments shown in Figure 1C and 1D, cells were grown in YPD liquid medium at 37 °C in log phase, or in YPD liquid medium at 37 °C for 3 d (post-exponential phase), and, to stimulate hyphal morphogenesis, in RPMI-10% bovine serum medium for 4–6 h at 37 °C.

### Plasmids.

Plasmid pCK73 was constructed by amplifying the *EFH1* ORF with primers VEC53F and VEC53R. The resulting fragment was digested with BglII and XhoI and cloned onto BglII, XhoI-digested pYB-ADH1pt [50]. Plasmid pCK74 and pCK75 were constructed by amplifying the *C. albicans HIS1* gene with primer AHISF3 and AHISR3, digesting the resultant fragment with AhdI and NheI cloning onto XcmI,XbaI-digested pCK73 or pYB-ADH1pt, respectively.

For gene deletions, *efh1*Δ::*ARG4*, *efh1*Δ::*HIS1*, *yhb5*Δ::*HIS1*, *yhb5*Δ::*ARG4*, and *yhb1*Δ::SAT flipper constructs were produced in several steps. First, *EFH1* sequences were amplified using primers pairs 53A and 53B2 or 53C2 and 53D2 followed by overlap PCR. The 1.1-kb fragment with *EFH1* upstream and downstream sequences flanking a unique PacI site with NotI sites on the ends was TOPO-cloned onto pYES2.1/V5-His-TOPO (Invitrogen) using manufacturer's protocols. The *YHB5* locus fragment was amplified with primers 44KOF1 and 44KOR1 and the *YHB1* locus fragment was amplified with primers YHB1F1 and yhb1R1; the fragments were TOPO-cloned as above.

To generate gapped or linearized plasmids, the *efh1* plasmid was digested with PacI, the *YHB5* plasmid with PacI, and the *YHB1* plasmid with HindIII. *HIS1* and *ARG4* markers were amplified from pGEM-HIS or pRS-ARG4ΔSpeI [44] with primer pair 53MF1 and 53MR1 (for *EFH1* knockout constructs) or 44MF1 and 44MR1 (for *YHB5* knockout constructs). The SAT1 flipper [36] was amplified with primers HB1NF1 and HB1NR1 for the *YHB1* knockout construct. Digested plasmids were cotransformed with appropriate PCR products into Saccharomyces cerevisiae strain EGY40 (*MATα ura3–1 his3–11 trp1–1 leu2–3*,*112*) [51]. Homologous recombination generated the desired constructs.

To construct pDRG-GFPS6, the *DRG1* promoter region [47] was amplified with primers XC3 and XC4 and cloned onto plasmid pXC31, a vector containing BamHI, NsiI, SalI, and EcoRV sites upstream of a promoterless *yEGFP* gene that was fused to the 3′ UTR of *ACT1* in pNUB1 (pNEB193 from New England Biolabs, with *C. albicans URA3* [47]).

To construct plasmid iSAT (encoding *SAT1* under control of the maltase promoter), the *SAT1* ORF was amplified with primers SATEC and SATR1 from pSFS2 [36]. The 3′ UTR of *ACT1* was amplified with primers SATAFU and ACT3x and fused to the *SAT1* fragment by overlap PCR. The resultant fragment was cloned onto vector pDBI52, generating a plasmid carrying *SAT1* under control of P*_MAL_* with a fragment of *C. albicans ADE2* for integration and the *C. albicans URA3* gene as a selectable marker.

### Animal models.

(1) Two germ-free piglets were inoculated orally with 10^9^ CFUs of C. albicans strain SC5314 and treated with 25 mg/kg body weight methylprednisolone and 15 mg/kg body weight cyclosporine daily as described previously [16]. Seven or 10 d post-inoculation, a piglet was sacrificed and organs were dissected and frozen in RNALater (Ambion) at −80 °C.

(2) Female Swiss Webster mice (18–20 g) were treated with tetracycline (1 mg/ml); streptomycin (2 mg/ml) and gentamycin (0.1 mg/ml) added to their drinking water throughout the experiment beginning 4 d prior to inoculation. C. albicans cells were grown for 24 h at 37 °C in YPD liquid medium, washed twice with PBS, counted, and adjusted to 2.5 × 10^8^ cells/ml. All strains were prototrophic. Mice were inoculated by gavage with 5 × 10^7^ C. albicans cells in 0.2 ml using a feeding needle. Colonization was monitored by collecting fecal pellets (produced within 10 min prior to collection) at various days post-inoculation and measuring C. albicans concentrations in the pellets by plating homogenates on YPD SA plates. Mice were sacrificed on various days post-inoculation and C. albicans concentrations in cecum contents, stomach contents, and homogenates of ileum, kidneys, liver, spleen, and tongue were measured by plating on YPD SA plates. Composite results from at least two experiments are shown.

(3) Intravenous inoculation of female CF1 mice (18–20 g; Charles River Laboratories) with 1 × 10^6^ C. albicans cells via the lateral tail vein was conducted as previously described [47]. Mice were observed twice daily after infection with C. albicans and were sacrificed when moribund.

(4) Competition experiments: To generate a marked WT strain, strain RM1000#2 His^+^ was transformed with plasmid iSAT, resulting in a prototrophic strain showing inducible resistance to nourseothricin. This genetic marker was used because it was expected that the maltase promoter would only weakly express the heterologous *SAT1* gene during growth of C. albicans in the mouse, thereby minimizing any potential deleterious effects due to *SAT1* expression.

For direct inoculation of the oral cavity by swabbing, the procedure was based on the previous work of Farah et al. [35] with minor modifications. Briefly, female BALB/c nude mice (5–7 wk, NCI) were given antibiotic water as described above. For inoculation, 1 × 10^8^ cells of C. albicans (mixture of two strains at a ratio of 1:1) were applied to a swab and introduced directly into the oral cavity. Colonization was monitored by swabbing the oral cavity and by collecting fecal pellets. Swabs were rubbed on YPD SA plates to assess colonization; fecal pellets were homogenized and plated. Intestinal colonization levels were comparable to those obtained by gavage.

To determine the ratios of the two different strains, the YPD plates were replica-plated to YPSnourseothricin and the ratio of nourseothricin-sensitive colonies (Nou^S^) to nourseothricin-resistant colonies (Nou^R^) was determined. At the later time points, some samples yielded few colonies (<10) and these samples were not included on the graph. The CI was obtained by dividing the ratio of Nou^S^/Nou^R^ colonies at time x by their ratio in the inoculum.

### RNA extraction.

Tissues from WT strain SC5314-infected IGB piglets, 7 or 10 d post-inoculation, were frozen in RNALater (Ambion) at −80 °C. For tongues and esophagus, the fungal cell–containing layer was cut from the tissue. WT C. albicans (SC5314 or DAY185) or mutant CKY138 (*efg1 cph1* double null) were inoculated into Swiss Webster mice by gavage. Three days post-inoculation, the contents of the cecum or ileum were recovered and frozen in RNALater at −80 °C. RNA was extracted from these samples using the Qiagen MIDI procedures with mechanical disruption using glass-zirconium beads, protease digestion, and on-column DNase treatment. In some experiments, samples were extracted with TRIzol, applied to a Qiagen column, and treated with DNase. For control piglet RNA, tissue was cut from the central portion of the tongue of an infected piglet and was not expected to contain significant numbers of C. albicans cells. RNA was extracted from this material as described above.

Reference RNA was extracted from cells grown in YPS medium at 34 °C in log phase, using Qiagen procedures with mechanical disruption and on-column DNase digestion. This RNA preparation was used as the reference sample for microarrays and for qRT-PCR.

### qRT-PCR.

10 μg of total RNA was converted to cDNA by incubation with Superscript II Reverse Transcriptase (Invitrogen) using an oligo dT primer. After incubation for 1 h at 42 °C, RNA was hydrolyzed and the reaction was stopped by addition of NaOH and EDTA to 0.16 N NaOH, 0.08 M EDTA, final concentrations. Following neutralization, cDNA was purified using Qiaquick columns (Qiagen) as described by the manufacturer, except that sodium acetate (pH 5.2) was added to the PB buffer to ensure an acidic pH. cDNA was quantitated by absorbance. Purified cDNA was stored frozen.

The following primer pairs (Table 4) were used to detect the indicated genes:

**Table 4:**
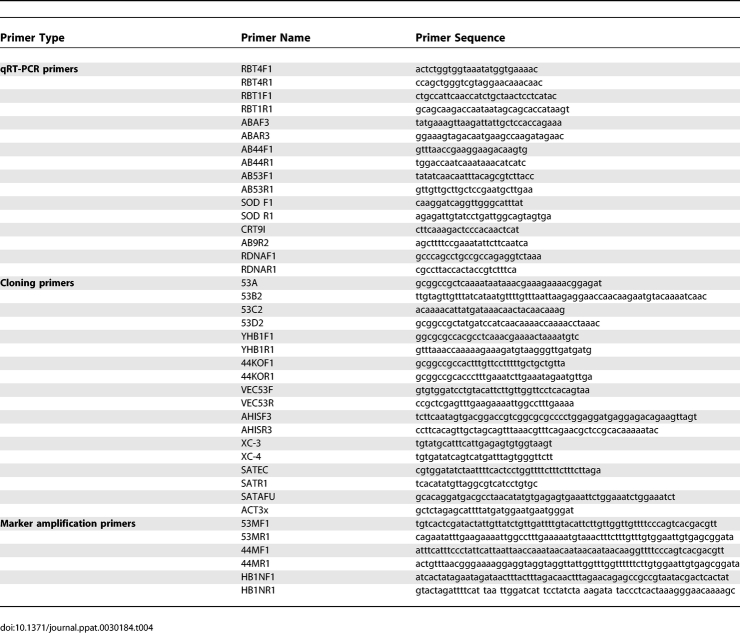
Primers

*EFH1*, AB53F1 and AB53R1; *YHB5*, AB44F1 and AB44R1; *ECE1*, CRT91 and AB9R2; *RBT1*, RBT1F1 and RBT1R1; *RBT4*, RBT4F1 and RBT4R1; *SOD3*, SODF1 and SODR1; *ACT1*, ABAF3 and ABAR3.

qRT-PCR was performed using SYBR green Mastermix and an ABI7700 instrument, according to manufacturer's protocols. All reactions were performed in triplicate. Melting curve analysis and/or agarose gel electrophoresis was performed following the reverse transcriptase PCR amplification to verify the presence of a single product. When RNA preparations not treated with reverse transcriptase were used as template, the primers failed to amplify products, or for *SOD3*, produced a signal nine cycles later than the signal obtained with cDNA, demonstrating that the amplified products reflected transcripts. Amplification of cDNA prepared from *efh1* null or *yhb5* null mutants with *EFH1* or *YHB5* primers, respectively, yielded no signal. cDNA prepared from piglet RNA yielded either no signal, or for *ECE1*, *RBT1*, and *RBT4*, a signal that was detected at least six cycles later than the signal obtained with a sample containing C. albicans. Therefore, the primers were detecting authentic *C. albicans-*derived transcripts.

PCR: Cecum contents were collected, and particulate material, including fungal cells, was pelleted at 3600*g* for 7 min and weighed. 70 mg of contents were extracted by bead beating in phenol-chloroform [49], followed by chloroform extraction and ethanol precipitation. The DNA was further purified using Qiagen DNeasy methods with RNase treatment. To determine the amount of C. albicans DNA, qPCR was performed in triplicate as above with rDNA primers (Table 4) designed from nonconserved regions of the rDNA sequence. The amounts of DNA are expressed in arbitrary units obtained using a standard curve of purified genomic DNA. The signals obtained from uninoculated mouse ceca were 100-fold lower than the geometric mean for WT C. albicans inoculated mouse ceca.

### Microarrays.

Labeled cDNA was prepared by reverse transcription of RNA with Superscript II Reverse Transcriptase (Invitrogen) using oligo-dT priming and Cy3- or Cy5-labeled dUTP (NEN). After incubation for 1 h at 42 °C, RNA was hydrolyzed and the reaction was stopped by addition of NaOH and EDTA to 0.16 N NaOH, 0.08 M EDTA, final concentrations. Following neutralization, cDNA was purified using Qiaquick columns (Qiagen) as described by the manufacturer, except that sodium acetate (pH 5.2) was added to the PB buffer to ensure an acidic pH. Dye incorporation was quantitated by absorbance. Cy3-labeled sample (40–60 pmoles) and Cy5-labeled reference (20 pmoles) were combined. Labeled sample cDNA was mixed with reference cDNA from log phase, laboratory-grown cells, and hybridized to C. albicans microarrays (version 5.1) containing 6,014 PCR fragments representing 91% of the ORFs in the C. albicans genome, as described previously [21]. Arrays were hybridized and scanned using Quantarray [21], and the results were analyzed visually or using Excel.

### Fluorescence microscopy.

Swiss Webster mice were inoculated with yEGFP-expressing C. albicans by gavage as above. At various days post-inoculation, mice were sacrificed and the contents of the ileum were recovered. Samples were filtered through 35-μm nylon mesh (Small Parts) and the filtrate was concentrated by centrifugation in an Eppendorf centrifuge for 1 min at maximum speed. Samples were observed using an Olympus BX60 microscope with GFP (excitation 460–490 nm, emission 515–700 nm) and YFP filters (excitation 500/20 nm, emission 535/30 nm) and photographed with the 60× objective.

## Abbreviations

CFU: colony forming unit
CI: competitive index
IGB: immunosuppressed gnotobiotic
OPC: oropharyngeal candidiasis
qRT-PCR: quantitative real-time reverse transcriptase PCR
qPCR: quantitative PCR
WT: wild type
yEGFP: yeast-enhanced green fluorescent protein
